# Comorbid psychiatric disorders in substance dependence patients: A control study

**DOI:** 10.4103/0972-6748.62265

**Published:** 2009

**Authors:** K. Shantna, S. Chaudhury, A. N. Verma, A. R. Singh

**Affiliations:** Department of Psychiatric Social Work, Ranchi Institute of Neuropsychiatry and Allied Sciences, Ranchi, Jharkhand, India; 1 Department of Psychiatry, Ranchi Institute of Neuropsychiatry and Allied Sciences, Ranchi, Jharkhand, India; 2 Department of Clinical Psychology, Ranchi Institute of Neuropsychiatry and Allied Sciences, Ranchi, Jharkhand, India

**Keywords:** Comorbidity, Mental disorders, Substance dependence

## Abstract

**Background:**

The purpose of this study was to investigate the comorbidity of mental disorders among a random sample of substance dependence patients from a psychiatric inpatients department and the general population.

**Materials and Methods:**

Comprehensive data was collected from inpatients with substance abuse/dependence and comorbidity of mental disorders at the Ranchi Institute of Neuropsychiatry and Allied Sciences (RINPAS) and from normal controls from the general population during the period January 2007 to May 2007.

**Results:**

The results show that the most prevalent comorbid disorders in substance dependence patients and substance abusers were depressive disorders.

**Conclusions:**

The majority of substance dependence patients suffered from comorbid mental disorders. Comorbidity needs to be taken into account when analyzing the relationship between substance dependence and depression and in planning treatment strategies for comorbid conditions.

Although the impact of co-occurring psychiatric disorders remains controversial, it is reasonably clear that alcohol-dependent individuals who meet the diagnostic criteria for one or more comorbid psychiatric disorders differ from those without comorbidity in many clinically relevant ways. Among alcohol-abusing and alcohol-dependent patients, prevalence rates for psychiatric comorbidity of between 57% and 84% have been reported (Lejoyeux and Marinescu, 2006). Mood disorders occ urring comorbidly with alcohol dependence are reported frequently. Many individuals with substance-use disorders also meet the criteria for major depression. Research on comorbid substance dependence and major depression was facilitated with the DSM-IV definitions of primary or independent disorders and substance-induced disorders, distinctions being based largely on timing. Substance-induced depression is defined as occurring during periods of substance use but exceeding the expected effects of intoxication or withdrawal from the substance used. Primary or independent major depression is defined as either predicting substance use entirely or occurring during periods of sustained abstinence. In one study, alcoholics with independent major depression were found to be more likely to attempt suicide than those with substance-induced depression. However, major depression that occurs before the onset of a substance-related disorder might relate differently to suicidal behavior than major depression that occurs during the period of sustained abstinence. The latter is more likely to occur while patients are in treatment (Davis *et al*., 2008). Studies of substance- misuse service populations have shown prevalence estimates for personality disorders ranging between 40% and 100% (Bowden-Jones, 2004). Personality disorders, especially antisocial personality disorders, are consistently associated with a worse long-term drinking outcome (Hunter *et al*., 2000).

## MATERIALS AND METHODS

Data was collected during the period January 2007 to May 2007 from inpatients of RINPAS and from the individuals in the general population. Demographic characteristics of the subjects are given in Table 1. The patient sample comprised of consecutively admitted patients 18 years old or older with dual diagnosis one of which was substance abuse/dependence. The matched control sample was drawn from the community by purposive sampling technique. Inclusion criteria required alcohol or substance use within 30 days of hospitalization (inpatients). Severely psychotic, medically ill or homeless patients were excluded. Of eligible patients, 100 randomly selected patients participated and were assessed by experienced interviewers.

**Table 1:** Age distribution of 200 male substance dependence patients

Age	Psychiatric patients *n* = 100 (%)	Normal controls *n* = 100 (%)
15-25	16 (16)	18 (18)
25-35	26 (26)	30 (30)
35-45	32 (32)	33 (33)
45-55	26 (26)	21 (21)

### Tools used

The following tools were administered:

#### Socio-demographic pro forma sheet

This self- made questionnaire contained questions on socio- demographic details, personal and clinical information, past and family history and habits of the subjects.

#### The brief psychiatric rating scale

The BPRS is a clinical rating scale widely used in psychiatric clinical practice. It is an 18-item scale measuring positive symptoms, general psychopathology and affective symptoms. Some items (e.g., mannerisms and posturing) can be rated simply on observation of the patient; other items (e.g., anxiety) involve an element of self-reporting by the patient. The BPRS is a clinician-rated instrument. Ratings are done after a brief (15-20 minutes) unstructured interview with the patient. Each item is rated on a 7-point scale (1 = ‘not present’ to 7 = ‘extremely severe’). Patient’s condition is judged at the time of interview, except for items numbered 2, 10, 11, 12, 15 and 16 for 3 days. When rating BPRS, it is important to allow unstructured sections in the clinical interview such that conceptual disorganization in the patient’s thought and speech and unusual thought content can be observed.

#### Michigan alcoholism screening test (Selzer, 1971)

The MAST is a 24-item screening instrument designed to identify and assess alcohol abuse and dependence. The MAST was originally developed as a structured instrument that was able to detect alcoholism in individuals and which could be administered by a range of clinicians.

#### The drug abuse screening test

The drug abuse screening test (Skinner, 1982) is a standard test that is used to determine if an individual is an addict. A score of 6 or more points indicates a drug problem. The DAST is the only method of identifying a potential drug problem. The DAST has been shown to have a high degree of internal consistency (coefficient *alpha* =.92), and factor analyses of inter-correlations among the DAST items have been interpreted as providing evidence that it measures a single dominant dimension of problems associated with drug abuse. Scores on the DAST are highly correlated with the frequency of use of a range of drugs, including cannabis, barbiturates, amphetamines and opiates other than heroin. The DAST attained 85% overall accuracy in identifying subjects who met DSM-III diagnosis and maximum sensitivity (96%) with a score of 6.

### Procedure

The interview tools were planned for the project. All patients were under the clinical management of a consultant psychiatrist. Face-to-face interviews were conducted. Interviews were also conducted on normal subjects from among the general population. Initially the socio-demographic and clinical details of the sample were taken from the patients and recorded in the socio-demographic data sheet. The rating scales were applied to the patients after the withdrawal symptoms had abated, usually after 2 weeks. Like the patients, the normal control subjects were also assessed by the assessment scales.

### Statistical analysis

The psychiatric patients were compared to normal controls by using the Chi-square test.

## RESULTS

In 81% of substance abuse/dependence patients, at least 1 diagnosis of comorbid mental disorder could be made. The most common disorders were depressive disorders, major depression, depressive disorder not otherwise specified, bipolar disorders and disthymia [Table 2]. Almost one third of the patients were diagnosed having major depression. One third of the major depressive disorders included psychotic features. Schizophrenia was diagnosed in 11% and bipolar disorder in 16% of the patients. Anxiety disorders were found in 6% of the cases; and personality and adjustment disorders in 9% and 13%, respectively. Suicidal ideation and suicide are very common phenomena in relationship with depression and substance dependence. Suicidal attempts were found in 10% of the patients; and suicidal thoughts and ideation, in 84% [Table 3]. There were some patients who did not reply on these issues. Regarding the methods used for attempting suicide, it was found that 3% attempted it by hanging; 2%, by taking drugs; 3% by jumping from high places; and 2%, by self-cutting [Table 4].

**Table 2:** Substance dependence and comorbid conditions

Disorders	Psychiatric patients *n* = 100 (%	Normal controls *n* = 100 (%)
Schizophrenia	11 (11)	0 (0)
Bipolar disorder	16 (16)	0 (0)
Depression	45 (45)	4 (4)
Anxiety disorder	6 (6)	9 (9)
Adjustment disorder	13 (13)	10 (10)
Personality disorder	9 (9)	10 (10)

**Table 3:** Suicidal attempts and ideation by substance dependence patients

	Psychiatric patients *n* = 100 (%)	Normal controls *n* = 100 (%)
Attempted suicide	10 (10)	2 (2)
Suicidal ideation	84 (84)	7 (7)
Not replied	6 (6)	51 (51)

**Table 4:** Suicide methods used

Method	Psychiatric patients *n* (%)	Normal controls *n* (%)
Hanging	3 (3)	1 (1)
Drugs	2 (2)	0 (0)
Jumping from high places	3 (3)	1 (1)
Cutting	2 (2)	0 (0)
Burning	0 (0)	0 (0)

## DISCUSSION

In the present study, the MAST and DAST questionnaires showed high specificity and high positive predictive value, but their sensitivity in patients is high because of high prevalence of substance dependence (cannabis, opiods and other forms of substance) in patients while in the general population, the diagnosis of alcohol dependence was rather high. The comorbid condition of schizophrenia was found in 11% of the substance dependence patients; bipolar disorder, in 16%; anxiety disorders, in 6%; personality disorders, in 9%; and adjustment disorder, in 13%. These findings support those of a few earlier studies. An analysis of the clinical profiles of 422 inpatients diagnosed with nonaffective psychosis (292, 295, 297 and 298 according to ICD-9) showed that a high percentage (42%) of these inpatients had psychotic pathologies and a history of substance abuse. These were predominantly young males. The percentage of schizophrenic patients with a history of substance abuse reached a level of 40% — a particularly relevant finding in view of the fact that substance abuse leads to worse prognostics (Vaz and Borrego, 2007). Fidalgo *et al*. (2008) reported that half of the heavy alcohol users presented with an additional psychiatric diagnosis.

Halikas *et al*. (1994) reported the most frequent comorbid disorders identified were phobic syndromes (27%), posttraumatic stress (18%) and major depression (16%). Lifetime disorders (73% of the sample) included antisocial personality (40% lifetime), phobia (34%), posttraumatic stress (27%) and major depression (23%); anxiety disorder preceded the first regular drug use in 76% of the clients with histories of anxiety disorder, but the first regular drug use preceded affective disorder in 65% of the clients with histories of affective disorders. In another study, Miller *et al*. (1996) reported on 6,355 voluntary admissions to 38 inpatient and 19 outpatient treatment programs affiliated with the Comprehensive Assessment and Treatment Outcome Research (CATOR) registry. The results revealed that 31% had received previous psychiatric treatment and 24% had received treatment specific to depression. On the basis of intake interview, 44% of clients had a lifetime diagnosis of major depression. Major depression was most likely to be associated with the use of opiods, stimulants and prescription drugs as specific drugs of abuse; with the number of drugs used; and with early onset of alcohol and marijuana use. Relapse at 1 year following treatment was equivalent for clients with or without diagnosis of major depression. Relapse was significantly associated with lower rates of lifetime depression for male cocaine users.

In the present study, among patients with substance abuse/dependence and comorbid psychiatric disorders, 10% of patients had attempted suicide while suicidal thought and ideation were reported by 84%. This is in agreement with a recent review which reported that nearly one third of patients with major depressive disorder also had substance use disorders, and the comorbidity yielded higher risk of suicide, greater social and personal impairment and other psychiatric conditions. Although the treatment of comorbid major depressive disorder and substance use disorders with medication is likely to be effective, the differential treatment effects based on substance use disorder comorbidity have not been systematically studied (Davis *et al*., 2008).

## CONCLUSION

We conclude from the study that substance abuse/dependence increases the risk of associated psychiatric disorders. The most common disorders are depression, bipolar disorder, schizophrenia and personality disorders. Depression is usually secondary to alcohol dependence. It is often associated with an increased risk of suicidal behavior.
